# Interim estimate of influenza vaccine effectiveness in hospitalised children, Hong Kong, 2017/18

**DOI:** 10.2807/1560-7917.ES.2018.23.8.18-00062

**Published:** 2018-02-22

**Authors:** Susan S Chiu, Mike Y W Kwan, Shuo Feng, Joshua S C Wong, Chi-Wai Leung, Eunice L Y Chan, J S Malik Peiris, Benjamin J Cowling

**Affiliations:** 1 Department of Paediatrics and Adolescent Medicine, Queen Mary Hospital and Li Ka Shing Faculty of Medicine, The University of Hong Kong, Hong Kong Special Administrative Region, China; 2 These authors contributed equally to this article; 3 Department of Paediatrics and Adolescent Medicine, Princess Margaret Hospital, Hong Kong Special Administrative Region, China; 4 World Health Organization Collaborating Centre for Infectious Disease Epidemiology and Control, School of Public Health, Li Ka Shing Faculty of Medicine, The University of Hong Kong, Hong Kong Special Administrative Region, China; 5 Center of Influenza Research, Li Ka Shing Faculty of Medicine, The University of Hong Kong, Hong Kong Special Administrative Region, China

**Keywords:** viral infections, influenza virus, vaccines and immunisation, vaccine effectiveness, test negative design, public health

## Abstract

We conducted a hospital-based test-negative study in Hong Kong to estimate influenza vaccine effectiveness (VE) for the winter of 2017/18. The interim analysis included data on 1,078 children admitted between 4 December 2017 and 31 January 2018 with febrile acute respiratory illness and tested for influenza. We estimated influenza VE at 66% (95% confidence interval (CI): 43–79) overall, and 65% (95% CI: 40–80) against influenza B, the dominant virus type (predominantly B/Yamagata).

Ongoing monitoring of influenza vaccine effectiveness (VE) provides important information to public health authorities, and supports evidence-based policy [1,2]. The test-negative study design is now used in many locations to provide timely estimates of VE [3,4]. The Hong Kong Special Administrative Region is a city with a population of 7.3 million, located on the south coast of China, and has a subtropical climate. We have been monitoring influenza VE in Hong Kong since 2009 [5-7] and present here the interim VE estimates in children for the 2017/18 winter season.

## Influenza activity in Hong Kong

**Figure 1:**
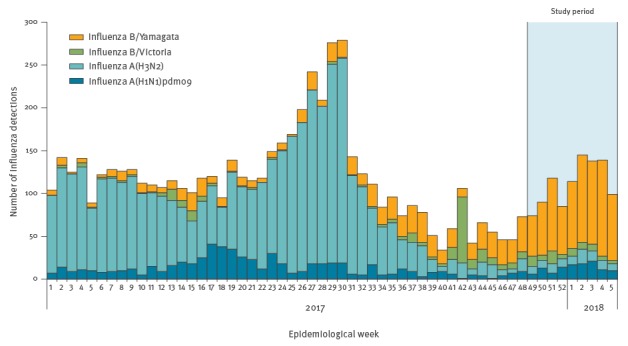
**L**ocal influenza activity as reflected by laboratory surveillance data, Hong Kong January 2017–January 2018 (n = 6,636)

Influenza circulates for most of the year in Hong Kong, with a winter peak in most years. In 2016/17, the winter influenza season was dominated by influenza A(H3N2) and had a moderate impact. That was followed by a very large summer influenza peak in July 2017 dominated by influenza A(H3N2), causing more than 400 laboratory-confirmed deaths [8]. Influenza activity had subsided through the autumn and at the end of 2017, influenza activity began to increase again, with influenza B/Yamagata predominating (Figure 1) [9]. In Hong Kong, most influenza vaccines are administered in October and November each year. Because of the lower influenza activity and the contemporaneous administration of vaccinations in October and November, we focus in this study on the period from 4 December 2017 through 31 January 2018.

## Influenza vaccine effectiveness

We conducted our study in two large hospitals in Hong Kong, Queen Mary Hospital and Princess Margaret Hospital [7]. In each hospital we enrolled children 6 months to 17 years of age who were admitted to the general wards of these hospitals with a febrile acute respiratory illness, defined as fever of ≥ 38 °C plus any respiratory symptom such as cough, runny nose or sore throat. Nasopharyngeal aspirates were obtained from all patients and tested for influenza A and B virus by direct immunofluorescence assay and reverse transcription PCR. Influenza vaccination history was recorded by research personnel in interviews with parents or legal guardians, using a standardised questionnaire, and compared with electronic medical records which contain some but not all influenza vaccinations. If parents showed any signs of being uncertain, we requested that they check their vaccination record and/or contact their private doctors if vaccination was done in the private sector. 

Vaccinated children were those who had received influenza vaccination for the 2017/18 season within the 6 months before admission in a regimen and dosage appropriate for their age and influenza vaccination history. Children who needed two doses of influenza vaccination but only received one dose, or who had received vaccination within 2 weeks before hospitalisation, were categorised as unvaccinated. The 2017/18 northern hemisphere formulation of trivalent and quadrivalent inactivated influenza vaccines were used during our study period.

We used conditional logistic regression to estimate the effect of influenza vaccination in reducing the risk of influenza-associated hospitalisation in children. To account for the potential confounding of this causal effect by age, we adjusted for age and age squared in the statistical model. We matched by epidemiological week to account for potential confounding by calendar time, since vaccination uptake increases through time and the risk of influenza varies over time. Influenza VE was estimated as 1 minus the adjusted conditional odds ratio (OR), multiplied by 100% [5,6]. Statistical analyses were performed in R version 3.4.0 (R Foundation for Statistical Computing, Vienna, Austria).

**Figure 2:**
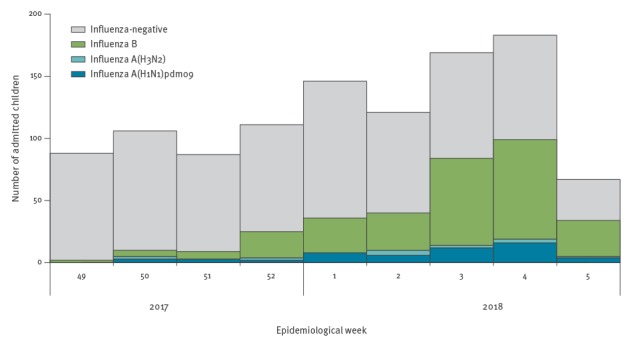
Timeline of recruitment of hospitalised children with acute respiratory illness who tested positive or negative for influenza virus by type/subtype, Hong Kong, 4 December 2017–31 January 2018 (n = 1,078)

From 4 December 2017 through 31 January 2018, we enrolled 1,078 hospitalised children. Of the 1,078 children, 339 (31.4%) tested positive for any influenza virus, and 271 of those 339 (79.9%) tested positive for influenza B (Figure 2). As local laboratory surveillance data indicated, almost all of the influenza B viruses circulating in Hong Kong in that period were B/Yamagata lineage viruses (Figure 1). 

The characteristics of the 1,078 children are shown in the Table.

**Table:** Comparison of hospitalised children who tested positive for influenza virus with children who tested negative for influenza, Hong Kong, 4 December 2017–31 January 2018 2018 (n = 1,078)

Characteristic		Influenza-positive (n = 339)		Influenza-negative (n = 739)		p value^a^
		n	%	n	%	
**Age group** ^b^	6 months–2 years	85	25.1	353	47.8	< 0.001
	3–5 years	123	36.3	226	30.6	
	6–17 years	131	38.6	160	21.7	
**Female**		158	46.6	323	43.7	0.410
Receipt of influenza vaccination^c^						
**Overall**		22	6.5	103	13.9	0.001
**By age group^d^**	6 months–2 years	5	5.9	28	7.9	0.679
	3–5 years	9	7.3	45	19.9	0.003
	6–17 years	8	6.1	30	18.8	0.003
**By type of vaccine** ^b^	Quadrivalent inactivated influenza vaccine	14	4.1	83	11.2	0.001
	Trivalent inactivated influenza vaccine	2	0.6	6	0.8	
	Both	0	0	1	0.1	
	Unknown	6	1.8	13	1.8	

^a^ p values estimated by chi-squared tests or Fisher’s exact test whenever appropriate.

^b ^Percentages are per total positive or total negative,

^c ^Receipt of influenza vaccination defined as receipt of a quadrivalent or trivalent inactivated influenza vaccine in an age-appropriate schedule within 6 months of hospital admission and at least 2 weeks before admission.

^d^ Percentages are per total positive or negative within the given age group.

Among the children who tested negative for influenza, 103 (13.9%) had been vaccinated, while 22 (6.5%) of the children who tested positive for influenza had been vaccinated.

We estimated that influenza VE was 65.6% (95% confidence interval (CI): 42.7–79.3) overall, 66.0% (95% CI: 3.4–88.0) against influenza A and 65.3% (95% CI: 39.5–80.1) against influenza B. VE was very similar for the quadrivalent vaccine that most vaccinated children had received, and we did not have sufficient data to estimate VE precisely for children who received the trivalent vaccine. 

There were 54 children younger than 8 years who had not been vaccinated in previous years and therefore required two doses of vaccine but had only received one dose at the time of admission. In our main analyses, we included these children as unvaccinated because one dose is not thought to provide full protection. In a sensitivity analysis we included these 54 children as vaccinated instead of unvaccinated, and estimated VE to be 58.1% (95% CI: 35.9–72.6) overall and 56.7% (31.4–72.7) for influenza B.

## Discussion

We found that VE against influenza B virus infections in children was moderate this winter in Hong Kong, consistent with the typical VE of inactivated vaccines against influenza B in children [10]. Our estimate was similar to the interim estimate of VE of 55% (95% CI: 38–68) against influenza B/Yamagata for the 2017/18 winter in adults in Canada where the trivalent vaccine was used [11], somewhat higher than the estimate of 41% (95% CI: 20–56) against influenza B in older children and adults in Spain again with the trivalent vaccine [12], and somewhat higher than the estimate of 42% (95% CI: 25–56) against influenza B in the United States [13]. Most vaccinated children in our study had received the quadrivalent formulation that contained a B/Yamagata component, rather than the trivalent formulation that did not. Our findings should be reassuring for northern hemisphere locations that are currently experiencing epidemics of influenza B/Yamagata, rather than the influenza A(H3N2) virus for which VE was reported to be very low [11]. In our study only 20% of influenza patients had influenza A, mostly A(H1N1)pdm09, and the estimate of VE against influenza A was less precise.

Influenza vaccination coverage in the children who tested negative for influenza is a proxy for vaccination coverage in the underlying population at risk of admission to hospital with influenza in Hong Kong, and was around 14% in this study, similar to 15% in the 2015/16 season [7], and somewhat higher than the average of 9% from 2009/10 to 2013/14 [5]. There is still considerable room for increasing the vaccination coverage in children. Since 2008, the local government has provided a subsidy for influenza vaccination administered by private-sector general practitioners to children between 6 months and 6 years of age and in October 2016, this was extended to children up to 12 years of age. The current subsidy is HKD 190 (ca EUR 20), and private general practitioners typically charge parents a consultation fee of around HKD 80–120 (EUR 8–12) in addition to collecting the subsidy. Children in low income families and children with underlying medical conditions are able to receive free vaccination from government clinics. One approach to increase vaccination coverage would be to introduce a school-based programme, and this could probably be implemented at a much lower cost per dose administered.

A limitation of our study is that we did not have lineage typing for the patients with influenza B, although local surveillance indicated that B/Yamagata was predominant. We did not have vaccine effectiveness data on adults or outpatients.

## Conclusion

We documented that influenza vaccination was associated with good protection against hospitalisation for influenza B virus infection in children 6 months to 17 years of age in Hong Kong in the winter of 2017/18. The majority of circulating influenza B viruses were B/Yamagata lineage.
